# Disintegrin-Integrin Binding for Attachment of Polymer Substrate to the Retina

**DOI:** 10.4172/2155-9570.1000752

**Published:** 2018-10-08

**Authors:** Alejandra Gonzalez-Calle, Rodrigo Brant, Bruno Diniz, Steven Swenson, Frank Markland, Mark S Humayun, James D Weiland

**Affiliations:** 1 Dr Allen and Charlotte Ginsburg Institute for Biomedical Therapeutics, USC Roski Eye Institute, University of Southern California, United States; 2 Department of Ophthalmology and Visual Sciences, Federal University of Sao Paulo, Brazil; 3 Department of Biochemistry and Molecular Medicine, Keck School of Medicine, University of Southern California, United States of America; 4 Department of Neurological Surgery, Keck School of Medicine, University of Southern California, United States of America; 5 Department of Biomedical Engineering and Ophthalmology, University of Michigan, United States of America

**Keywords:** Disintegrin integrin, Visual impairment, Method vicrostatin Retina, Retinal prostheses, Novel attachment

## Abstract

**Objective:**

We propose a novel attachment method for retinal tissue that utilizes silicone modified with bioactive molecules.

**Design:**

This is an experimental study divided into an *in vitro* section performed in cadaveric pig eyes and an *in vivo* section performed in rabbits.

**Subjects:**

During *in vitro* experiments 36 cadaveric pig eyes were used. During *in vivo* experiments 4 rabbits were used.

**Methods:**

Different types of silicone went through a laser irradiation process to determine if binding sites for disintegrins could be created. Laser treated silicones that showed disintegrin binding were evaluated with *in vitro* testing for retina-silicone attachment. The best silicone binding *in vitro* was implanted into a rabbit’s eye after a full vitrectomy was performed. Post-operative exams were done every two weeks to evaluate placement, attachment and sterilization method. After three months animals were euthanized and eye was enucleated for histology analysis.

**Main Outcome Measures:**

Attachment strength between silicone-disintegrin-retina, and signs of endophthalmitis during *in vivo* studies for biocompatibility purposes.

**Results:**

A technique to successfully lase and produce an active area on the silicone surface was described. Scanning electron microscope (SEM) images were evaluated to assess physical ablation/debris field area on the surface, definition of edges, evenness, and symmetry of the lased area allowing us to select MED 4800 silicone family for further testing. Cell culture experiments showed disintegrin binding to the silicone active area. *In vitro* experiments with cadaveric eyes were performed to test retina-silicone attachment. MED 4860 showed strongest attachment to the retina and it was used during *in vivo* experiments. A sterilization protocol was tested and proved to be reliable for bioactive materials. Disintegrin coated silicone showed attachment in 2 of 4 rabbits during the 3-month implant period. The adhesion was persistent until reversed with plasmin. All rabbits were implanted for 3 months regardless of attachment, to test the feasibility of the sterilization method. None of the rabbits developed any type of eye infection during the implant period.

**Conclusion:**

We successfully lased and produced an active area on the silicone surface to allow disintegrin-silicone binding. Different silicones interact differently with the laser energy, and this is reflected in the strength of the silicone-disintegrin-retina attachment.

## Background and Objective

Visual impairment is a major concern due to an overall aging population and the increased risk of accidents, loss of independence, and depression in visually impaired patients [1]. New devices are being developed as treatments for conditions such as glaucoma, age related macular degeneration (AMD) and retinitis pigmentosa (RP). Glaucoma is a condition in which the intraocular pressure (IOP) increases, causing damage to the optic nerve and blindness if not treated [2]. A glaucoma drain has been designed to help decrease the IOP *via* an adjusTable valve that decreases the resistance to aqueous humor outflow by creating a supplemental flow pathway [3]. Dry eye is a disorder of the pre-ocular tear film in which the eye cannot produce tears properly to keep the eye moist or the tears produced are not the right consistency, so they evaporate faster. If left untreated, pain, corneal ulcers and loss of vision can occur [4]. For both glaucoma and dry eye, the most common treatment is eye drops applied to the cornea, requiring compliant patients to self-administer the drugs. Consistent drug delivery is a critical factor for this treatment to be successful and this is why drug delivery systems have been developed, to help patients by delivering nano-liter dosages of medication every hour, day, or month as needed and lasting for several months before replacement or refill [5].

Aged Related Macular Degeneration (AMD) [6] and Retinitis Pigmentosa (RP) [7] affect the photoreceptors layer, causing the retina to lose its ability to translate light into electrical signals, causing profound vision loss and blindness in the late stages. Retinal prostheses [8] are used to treat these conditions by electrically stimulating the remaining healthy retinal neurons (bipolar and ganglion cells) [9]. All of the aforementioned devices will benefit from a technology that allows easy, reversible attachment of a device to eye tissue. While sutures and tacks are commonly used, other options of attachment should be explored that are less traumatic to tissue. In this study we propose a novel attachment method based on disintegrin-integrin binding and a process for creating silicone modified with bioactive molecules.

Integrins are heterodimeric cell surface receptors found throughout the body that mediate cell adhesion and migration by interacting with proteins in the surrounding extracellular matrix [10]. Integrins exist in three conformational states: an inactive or low affinity state, a primed or activated high affinity state, and a ligand bound or occupied state [11]. Some activated integrins such as the αv and α5 members, are not displayed by quiescent tissue, but play an important role in processes including attachment, invasion and angiogenesis [12,13]. Disintegrins are disulfide-rich peptides, many of which contain an Arg-Gly-Asp (RGD) sequence that binds to integrins on the surface of cells [14]. Contortrostatin (CN) a homodimer [15] and vicrostatin (VCN) a monomer [16] are two disintegrins that contain an RGD tripeptide motif that binds with high affinity to specific integrins (αIIbβ3, αvβ3, α5β1, and αvβ5) [17]. Contortrostatin (CN) was isolated from the venom of the copperhead snake and vicrostatin (VCN) is derived from CN and was produced in the research laboratory using recombinant DNA technology. In humans, integrin subunits (α1, α2, α3 and β1) are present in the Muller cell [18] foot end processes, which forms the inner limiting membrane of the retina. Previous experiments have shown that VCN and CN bind to these integrins [19,20].

Binding of disintegrins to silicone is made possible by laser treating the silicone surface [19]. Laser irradiation results in selective surface decomposition of silicone that preserves the (inorganic, Si-O) backbone structure of the polymer and eliminates its organic part (C-radicals). Use of a UV laser source at 248 nm (corresponding to 5.0 eV photon energy) limits photon absorption to Si-C bonds. The end product of that decomposition is a polymeric chain (i.e. poly-silica) that is formed of Si-O monomers. Both the irradiated surface and lateral debris areas are electrically “active”, being negatively charged due to the two dangling bond electrons that are attached to each Si atom along the chain structure of poly-silica. These Si-dangling bond electrons can be utilized in attaching CN and VCN covalently to the surface of the silicone [19,21]. Here, we report on experiments that evaluated silicone-VCN material applied to retinal tissue.

## Material and Methods

### Silicone-disintegrin process

Experiments on selective surface irradiation were performed on several commercially available formulations of silicone to investigate whether an active area could be created. These experiments studied both the physical ablation/debris field area on the surface, and the degree of disintegrin binding to the modified surface area under different laser processing conditions (patterns, depth, and size of the lased area). The NuSil MED-4800 family (4810, 4830, 4840, 4850, 4860) and other silicones (NuSil MED 4286a DowCor WL5150) were tested.

To modify the silicone surface, an ATL ProMaster Excimer Laser (ATL Lasertechnik GmbH, Wermelskirchen, Germany) was used with a 248 nm wavelength under KrF medium. The laser beam was focused on the sample through a given geometrical shape.

Table 1 shows the settings that showed a consistent debris field area on the surface, even depth and clean edges, as judged by visual inspection. These parameters were used during the rest of experiments.

CN and VCN were evaluated for their binding affinity to the active silicone surfaces and for their ability to support cellular growth. After lasing, silicone samples were submerged in the disintegrin solution, incubated overnight, washed in phosphate buffered saline (PBS), and separated into two groups. In Group 1, attachment strength to retina was measured *in vitro* to determine if the disintegrins were tightly bound to the laser modified silicone. In Group 2, ovarian cancer cell line OVCAR-3 was plated on the sample and images recorded every 6 h (up to 48 h post incubation) to measure the ability of the disintegrins to promote cellular attachment and growth The cells were transfected with green fluorescent protein (GFP) to aid visualization.

### Silicone sterilization

Silicones were sterilized using ISO 11135 Ethylene Oxide (EtO) protocol for medical devices. To make sure EtO was bioactive material-friendly, we tested two different protocols to verify we were not causing any modifications of the biological materials:

1) Silicones were sterilized with EtO before lasing process. After sterilization, the lasing process was performed in a clean environment, followed by exposure to disintergin solution
2) Silicones were lased, exposed to the disintegrin solution overnight and then sterilized with EtO.

Both methods were evaluated by measuring disintegrin-retina attachment, as described below.

### *In vitro* experiments

Cadaveric pig eyes (Sierra Medical Supply,Inc, Whittier, CA, USA) were used for *in vitro* experiments. The pigs were all healthy and aged between 5-7 months (n=36). The time between animal sacrifice and the start point of our experiment was less than twelve hours. Diluted (Dulbecco’s PBS) plasmin (EMB Biosciences, Inc) solution (0.15 ml) was injected into the vitreous cavity 3 days before VCN-silicones were implanted, to create a posterior vitreous detachment (PVD) and facilitate removal of the vitreous. The eye was refrigerated at 4 Celsius after injection of plasmin. After 3 days, the eye was pinned to a foam board and three incisions, similar to standard vitrectomy, were made in the sclera to place the infusion line, the vitrectomy cutter and the intraocular light. A 20-gauge instrument (Stellaris 20 g vitrectomy pack) was used. The vitreous was then completely aspirated (Stellaris, Bausch and Lomb, Rochester, NY, USA). After vitrectomy was completed, the cornea of the pig eye was removed to facilitate epiretinal placement of the VCN-silicone samples. Samples were held in place for 20-30 seconds, and then pulled off using forceps. The surgeon did not have knowledge of what type of silicone or disintegrin was used. The surgeon then estimated the relative strength of attachment in each case.

### Surgical procedure-*In vivo* experiments

Adult pigmented rabbits (Irish Farms, Norco, CA), ~3 months old were used for all experiments. Implantation of the VCN-silicone was performed in the left eye of each animal (n=4). After VCN-silicone implantation, animals were kept for a period of three months and then euthanized. All animals were maintained on a daily 12 h light/dark cycle. All procedures were in conformance with the Guide for Care and Use of Laboratory Animals (National Institutes of Health). The University of Southern California Institutional Animal Care and Use Committee reviewed and approved all procedures.

Prior to surgery, 0.15 ml diluted plasmin solution was injected into the vitreous cavity while the eye was observed under a surgical microscope. This solution helps liquefy the vitreous and makes it easy to perform vitrectomy without causing retinal detachment. Thorough removal of the vitreous allows clean exposure of the epiretinal surface, which has binding sites for disintegrins. A week after injecting plasmin, the VCN-silicone implantation surgery was performed.

The pupil was dilated with three drops each of 1% tropicamide and 2.5% phenylephrine. Three incisions were made in the sclera to place the infusion line, the vitrectomy cutter, and the illuminating fiber optic probe; 25 gauge instruments were used (Stellaris, Bausch and Lomb). A vitrectomy was then performed. After initial aspiration of the liquefied vitreous, triamcinolone acetonide (Triesence) suspension (40 mg/ml) was injected into the eye to stain residual vitreous bound to the epiretinal surface. After staining, the residual vitreous could be visualized and removed, leaving a clean epiretinal surface. The VCN-silicone was then inserted into the vitreous cavity with the disintegrin surface facing the retina; it was placed on the epiretinal surface, close to the optic nerve. Care was taken to avoid contact between VCN-silicone and blood, as this would contaminate the disintegrins. Placement was evaluated under a surgical microscope. Once the VCN-silicone was properly positioned, it was held in place for 20-30 seconds to promote disintegrin-integrin binding. After implantation, the infusion line and light were removed and incisions were sutured with 6-0 vicryl.

Post-operative exams were done every two weeks for a three-month period. If the optical path was clear, fundus images and Optical Coherence Tomography (OCT) images were taken to evaluate placement, attachment of the VCN-silicone, and damage of the retina. Fluorescence Angiography (FA) images were taken to evaluate the preservation of the vasculature of the retina under and around the implant. The eye was observed for signs of endophthalmitis, to test the sterilization method. After three months animals were euthanized and eye was enucleated for histology analysis.

## Results

### Silicone-disintegrin process

**Figure 1:**
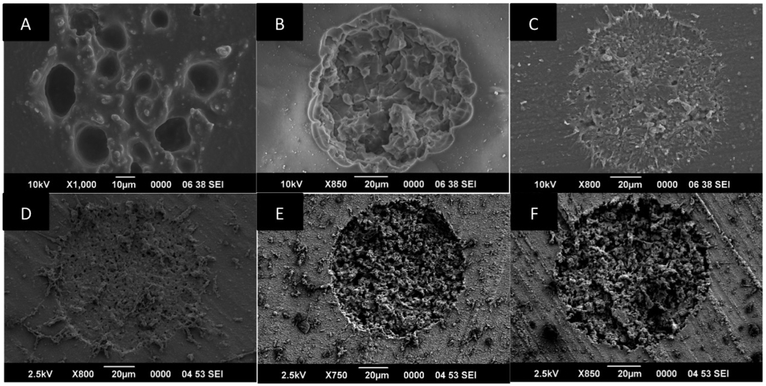
Different types of silicones were lased to evaluate if an active area could be created. The parameters in Table 1 were used during all the experiments performed. SEM images were taken of each type of silicone after lasing process. The silicones shown in this image are A) MED 4286 B) WL5150 C) MED4830 D) MED4840 E) MED4850 F) MED4860. MED 4850 and MED 4860 have edges that are clean and defined, uniform ablation area, and a large debris field.

Six different types of silicone were evaluated to see if an active area could be created. Silicones were lased and scanning electron micrograph (SEM) images were taken from each sample (Figure 1). We visually assessed each sample to judge the debris field area on the surface, the amount of material removed, definition of edges, evenness, depth, and symmetry of the lased area. Figure 1 shows how MED 4850 (Figure 1E) and MED 4860 (Figure 1F) have edges that are clean and defined, uniform ablation area, and a large debris field. Other formulations like MED 4286 (Figure 1A) and WL5150 (Figure 1B) shows poor definition on the edges, poor debris field area, and unevenness in the lased area. MED 4830 (Figure 1C) and MED 4840 (Figure 1D) show clean and defined edges, however, although the ablation area is even it lacks depth and only a small debris field is observed.

**Figure 2:**
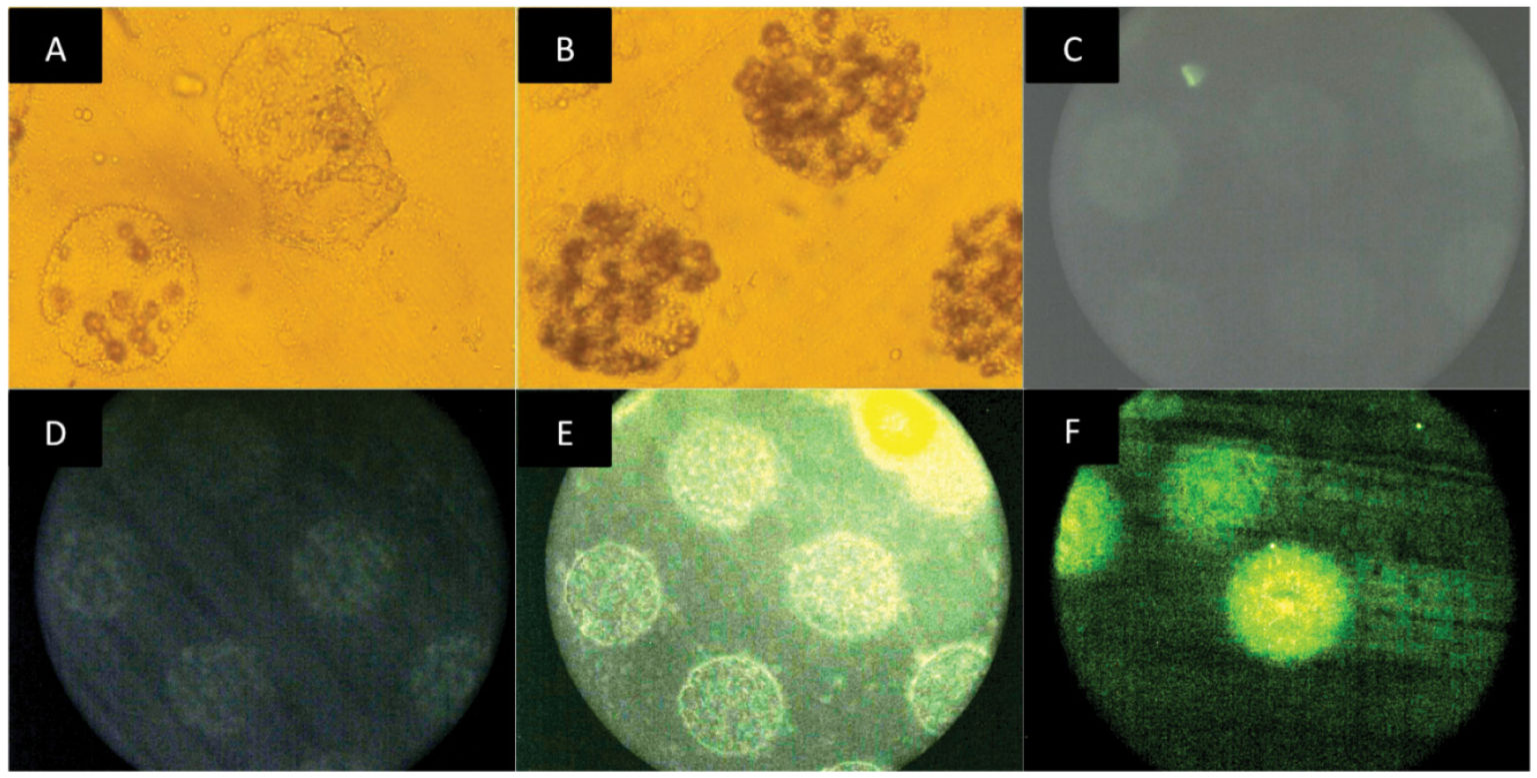
Images taken to evaluate disintegrin binding (VCN reported in these pictures) and cell growth on silicones after lasing process and disintegrin exposure: A) MED 4860 six hours after plating cells and B) MED 4860 48 hours after plating cells shows cell growth by light microscopy on the lased area but not on the unlased areas that were also exposed to disintegrin. C) MED4830, D) MED4840, E) MED4850, and F) MED4860. C-F were exposed to OVCAR -3 and cell growth is evaluated by fluorescence. MED 4830 to MED 4860 support cell growth but MED 4860 is superior in supporting biological function of the disintegrins (increase in fluorescence observed due to increased OVCAR3 cell growth).

Twelve more samples (2 for each type of silicone mentioned above) were lased and treated with disintegrins to evaluate the degree of disintegrin binding to the modified surface area (Figure 2). By visual inspection, each sample was evaluated for growth of OVCAR3, human ovarian cancer cells that display integrins αvβ3 and αvβ5 and are fluorescent; increased fluorescence indicates more cell growth and a better substrate for integrin-disintegrin binding. MED 4860 was imaged six hours post plating (Figure 2A) and 48 h after incubation (Figure 2B), showing a difference in cell growth between the two time points. At 48 h we can observe how cells grew only on the putative location of disintegrins (as defined by lasing). Unlased, control silicone showed no cell growth 48 hours after incubation (images not shown). In MED 4830 (Figure 2C), and MED 4840 (Figure 2D) some cell growth was observed 48 hours after incubation but MED 4850 (Figure 2E) and MED 4860 (Figure 2F) had a confluent layer of cells at the same period of time. MED 4860 was superior in supporting biological function of the disintegrins, as indicated by the highest level of fluorescence. MED 4286, MED 4810, WL5150 did not show cell growth during the 48 h period (images not shown). Table 2 shows a summary of the results obtained in this experiment.

### *In vitro* experiments

*In vitro* experiments were performed to evaluate attachment. Four types of silicone were used, based on the results above: MED 4830, 4840, 4850 and 4860. Eight (two of each type of silicone) samples were lased and immediately placed in disintegrin solution (CN or VCN), incubated overnight, and washed in phosphate buffered saline (PBS) before being implanted in a cadaveric pig’s eye. After vitrectomy and corneal removal, samples were given to a retina surgeon; one sample per eye was used. The surgeon held silicone in place for 20-30 seconds to promote adhesion, then pulled it off with forceps and reported the observed strength of attachment.

Table 3 Show a summary of the results obtained in this experiment. MED 4830 showed no observable attachment, MED 4840 showed very poor attachment and it was pulled off very easily from the retina, MED 4850 showed a slightly better attachment than MED 4840 but it could be pulled off the retina with moderate force, MED 4860 showed a strong attachment to the retina, and when surgeon tried to remove the sample the retina stayed attached to the sample and detached from the eye. There was no observable difference between CN-silicones and VCN-silicones.

### Sterilization protocol

For sterilization testing, we continued testing the last four different types of silicones that showed disintegrin binding (MED 4830, MED 4840, MED 4850 and MED 4860). Twenty-eight (seven of each type of silicone) samples were used to evaluate sterilization. Samples were divided in four different groups: Group 1-Eight samples (2 of each type of silicone) were lased and immediately placed in disintegrin solution (1 in CN and 1 in VCN), incubated overnight, and washed in phosphate buffered saline (PBS). Group2-Eight samples (2 of each type) were sterilized using clinical ethylene oxide (EtO) protocol, and then lasing and disintegrin exposure was performed.

Group 3-Eight samples (2 of each type) were lased, placed in disintegrin solution, incubated overnight, washed in PBS, and then sterilized using EtO.

Group 4-Four samples (one of each type) served as control with no sterilization or lasing. Samples were washed in PBS before implantation.

Samples were tested in cadaveric pig eyes. After vitrectomy was completed, and the cornea removed for easy placement, samples were given to the surgeon; one sample per eye was used (n=28). The surgeon held silicone in place for 20-30 seconds and then removed it with forceps and reported his assessment of the strength of attachment. Table 4 shows a summary of the results obtained during this experiment. Non-sterile samples (Group 1) and samples lased after sterilization (Group 2) showed the same results reported above where MED 4830 shows no attachment to the retina, MED 4840 and MED 4850 shows poor attachment to the retina and with MED 4860 a strong attachment to the retina was observed. No difference between CN- Silicone and VCN-Silicone was reported. Samples sterilized after lasing procedure (Group 3) and control group (Group 4) showed no attachment to the retina for any of the silicone groups and no difference between CN-Silicone and VCN-Silicone.

Based in the results reported above MED 4860 and the group 2 sterilization method were used for *in vivo* experiments. VCN-silicone was selected because no difference between CN-silicone and VCN-silicone was reported in any of the experiments and because VCN is a recombinant disintegrin that is more easily obtained and the likely path towards translation to a medical device.

### *In vivo* experiments

Adult pigmented rabbits, ~3 months old were used for all experiments (n=4). Two out of the four rabbits implanted showed attachment (Rabbit 1 and 2) of the silicone to the surface of the retina during the entire evaluation period. Rabbit 3 and 4 did not show silicone-retina attachment but they were evaluated for feasibility of the sterilization method, since we left the VCN-silicone sample in the vitreous cavity. Rabbits 1, 3, and 4 were observed for 2 months and rabbit 2 was observed for three months.

**Figure 3:**
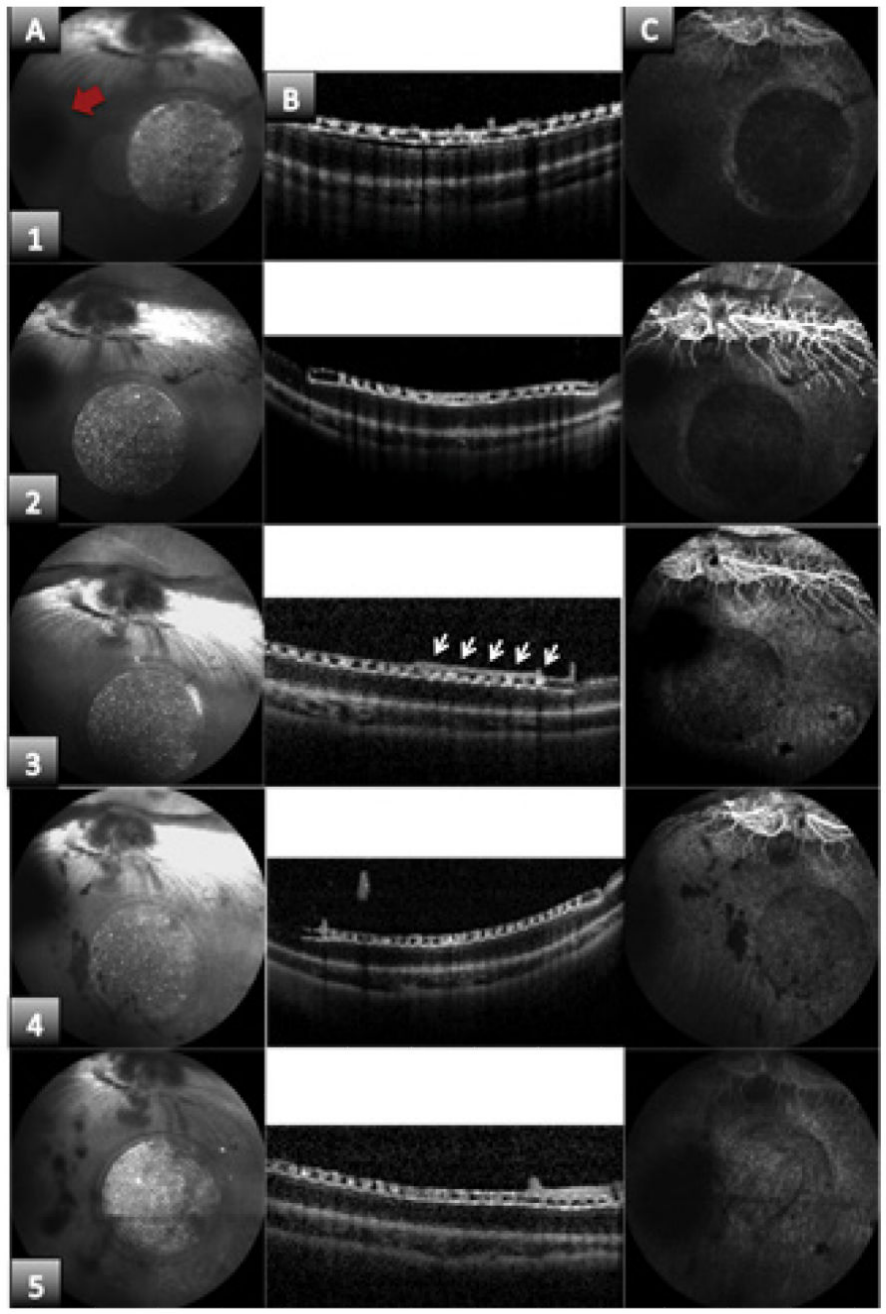
Images were taken to evaluate disintegrin binding in silicones after lasing process, retina-silicone attachment and feasibility of sterilization method. Three images were taken. Column A shows a fundus image of the retina with the silicone sample attached to it. Column B shows an OCT image, which represents a cross section of the retina where the sample is attached and Column C shows a Fluorescein Angiography (FA) image to evaluate retinal circulation. These three types of images were taken at 5 different time periods after implantation: Row 1) One week, Row 2) two weeks, Row 3) 5 weeks, Row 4) 2 months and Row 5) 3 months. During the evaluated period of time, silicone sample remained attached, no infection or inflammation was observed, and no vessel leakage was detected. Red arrow shows a cataract formation.

None of the rabbits developed any signs of eye infection during the evaluation period. Rabbit 1 was evaluated every two weeks for a two-month period with fundus images. The VCN-silicone sample was placed very far from the optic nerve, so Optical Coherence Tomography (OCT) images could not be taken. Rabbit 1 showed attachment of the silicone to the retina surface in every evaluation during the two-month period. Rabbit 2 was evaluated every two weeks for a three-month period using fundus images and OCT images (Figure 3). At two weeks after implantation (Figure 3: 1) VCN-Silicone attachment to the retina in OCT image was observed (Figure 3: 1B); fundus image (Figure 3: 1A) shows attachment of the silicone to the retina and a small cataract developing (black shadow shown with red arrow) caused during surgery. Fluorescence Angiography (FA) image (Figure 3: 1C) shows well preserved vasculature and no evidence of leakage or pooling of blood. At five weeks after implantation (Figure 3: 3) VCN-Silicone attachment to the retina in OCT image was observed (Figure 3: 3B) and a small tissue layer was observed growing on top of the VCN-Silicone sample (arrows); fundus image (Figure 3: 3A) shows attachment of the silicone to the retina and cataract sTable. FA image (Figure 3: 3C) shows no vessel leakage. At 3 months after implantation (Figure 3: 5) VCN-Silicone attachment to the retina in OCT image was observed (Figure 3: 5B), and tissue layer on top of the VCN-Silicone can still be observed; fundus image (Figure 3: 5A) shows attachment of the silicone and no progression of cataract. FA image (Figure 3: 5C) shows no vessel leakage. No signs of infection was observed throughout implant period. Summary of results are reported in Table 5.

**Figure 4:**
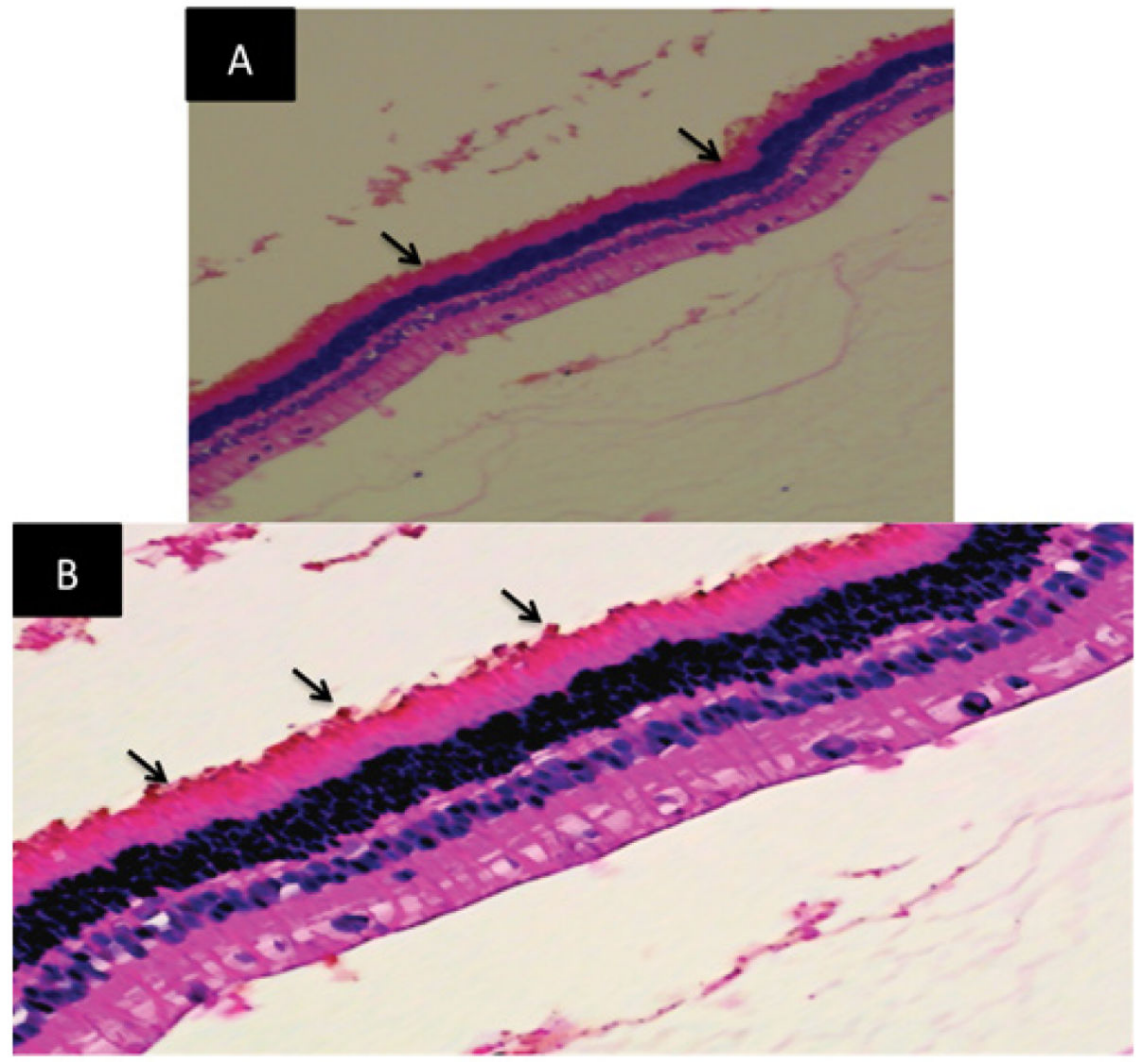
Histology images taken from rabbit 2. (A) Arrows show area where sample was placed. (B) Zoomed image of area between arrows in image A. No significant damage is recorded in these images. Image B shows some red blood cells on the retinal surface (arrows point to the red blood cells).

After a two and a three-month period for rabbit 1 and 2 respectively, animals were anesthetized and plasmin was injected in the vitreous cavity near the sample to detach sample from retina surface. Plasmin allowed us to detach silicone sample from the retina without causing any damage to the retina. Plasmin was diluted in Dulbecco’s PBS and 0.15 ml were injected. When plasmin was injected, one side of the silicone sample lifted up from the retina but the other side stayed close to the retina, due to fibrosis growing on top of the sample. The sample was removed when it was visibly detached from the retinal surface. Retinal detachment was not observed after sample removal. The eye was then enucleated and the tissue fixed for processing. Staining with Hematoxylin and Eosin (H&E) shows no significant damage to the retina in the area where sample was placed. Histology shows some red blood cells on the surface of the retina that could have been caused by the surgeon when removing the fibrosis to release the sample (Figure 4).

## Discussion and Conclusion

**Figure 5:**
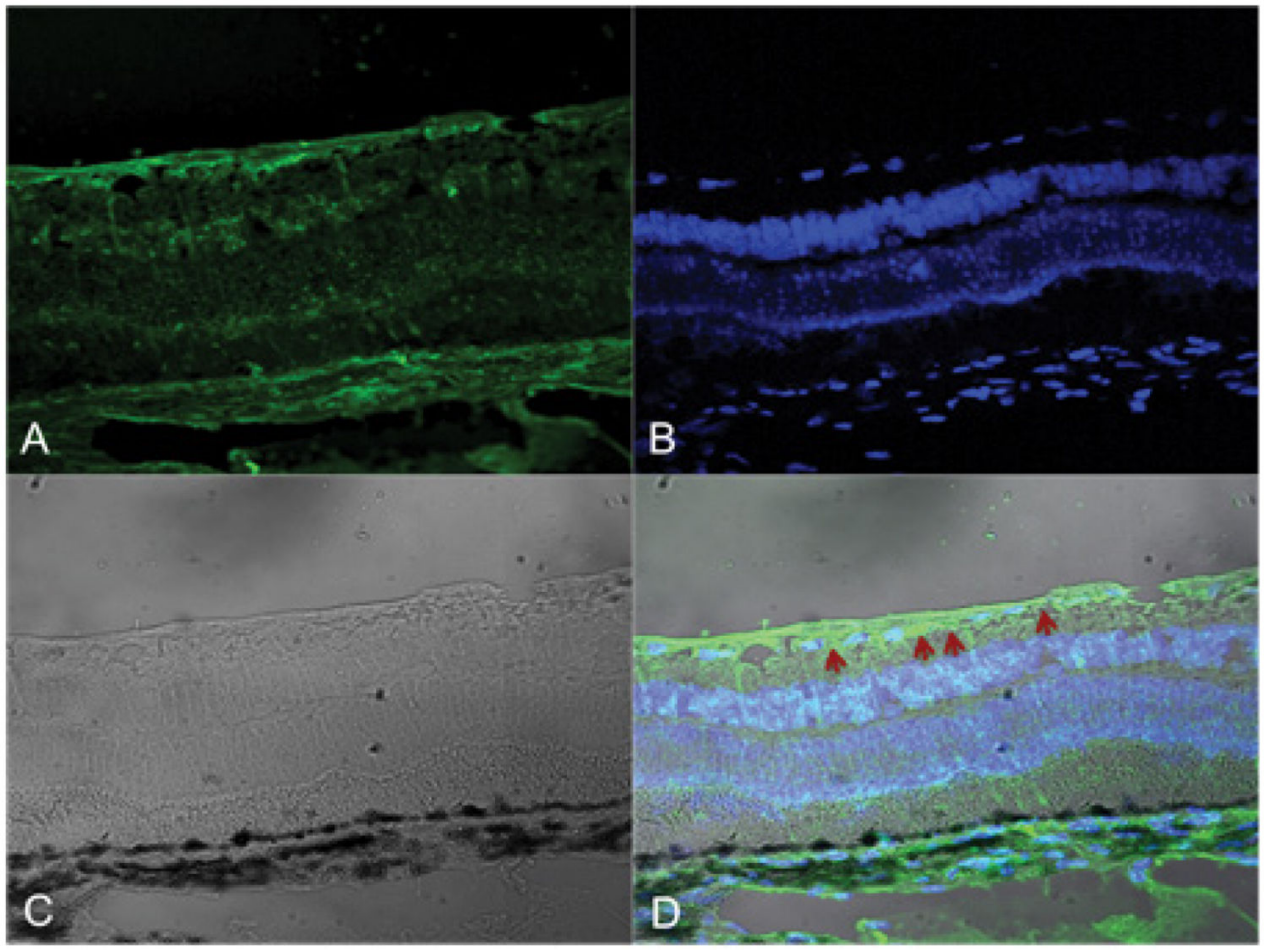
Rabbit retina integrinβ5. Panel A. Green fluorescence stains β5 integrin. Panel A demonstrates presence of β5 integrin in the ILM of the rabbit retina. Panel B. Blue fluorescence is DAPI counter staining of the nuclei of the neurons. Panel C. Grey image is photomicrograph. Panel D is an overlay of all three images. Red Arrows shows the ILM of the rabbit retina.

In this communication we have described a technique that allows us to use disintegrin-integrin binding as a novel attachment method for retinal tissue. Integrins are α/β heterodimeric glycoproteins that are expressed on the surface of mammalian cells [10] and are involved in the regulation of cell growth and survival [22]. So far 18 α- and 8 β-subunits have been described for integrins in human cells; they mediate a wide range of cellular functions, including adhesion [23]. Disintegrins are disulfide-rich, RGD-containing peptides that bind to integrins on cells. CN, a homodimeric disintegrin originally isolated from southern copperhead venom binds to integrins of the β1, β3 and β5 subclasses, including receptors for fibronectin (α5β1), vitronectin (αvβ3, αvβ5), and fibrinogen (αIIbβ3) [14]. VCN, a recombinant monomeric disintegrin based on the sequence of CN, retains the same integrin binding characteristics as CN, but is available in unlimited amount. For the integrin-disintegrin approach to work, integrins must be present in the target issue (retina). Integrin subunits α4, α5 and α5 (and most likely others as well) are present in the inner limiting membrane (ILM) of the retina in rabbits and dogs (Figure 5) proving a rich substrate for integrin-disintegrin binding.

We have described a technique to successfully lase and produce an active area on the silicone surface that will allow disintegrin binding. Our study evaluated different types of medical grade, commercially available silicones and how they interact with laser processing. Significant differences were noted based on the type of silicone used. These differences affected the silicone-VCN binding and the strength of the silicone-VCN-retina attachment. This knowledge will be useful for choosing a stronger or weaker attachment, which could be used to control silicone-VCN-retina binding depending on the particular needs of the device. Future experiments should be directed towards understanding the fundamental physical mechanisms that determine binding strength.

Different approaches have been utilized to attach the retina prosthesis to the retina as an alternative to a retinal tack. To test magnetic fixation, researchers implanted one magnet in the suprachoroidal space, and a second magnet on the back of an intraocular component. This arrangement was used to successfully position a retinal prosthesis epiretinally for a 6 week period, maintaining the position reliably, but causing trauma to the retina on the edges of the device [24]. Other adhesive materials such as fibrin glues, photocurable glues and hydrogels have been tested, proving hydrogels were much more adherent to the retina than the other bioadhesives [25]. Mucoadhesive materials such as chitosan, thiomers, boronate-containing polymers, liposome-based mucoadhesive formulations and acrylic-based polymers have shown potential for tissue adhesion [26]. Further studies will have to be performed to evaluate their applicability for specific applications like retinal prosthesis adhesion.

To consider a device for human use, sterility is an absolute requirement. The main issue with the most common FDA-approved terminal sterilization techniques is that they are not bioactive-material-friendly, causing chemical modifications of biological materials [27]. During our study a sterilization protocol was tested and proven to be reliable for bioactive materials. This involved sterilization of the silicone device before lasing using a clinical ethylene oxide protocol and then doing the lasing and disintegrin attachment under sterile conditions. Other protocols like supercriticial CO_2_ (scCO_2_) in the presence of H_2_O_2_ have been studied and have proven to be reliable for sterilization of bioactive materials [27].

In both our results and the results reported by Rowley [19,20], VCN bound tightly to the retina of the cadaveric pig eyes when MED 4860 was used. *In vitro* experiments by Rowley [20] with enucleated porcine eyes showed that with good technique and practice, the Silicone-VCN (CN)-Retina bond forms very rapidly (within 10 seconds) and is very strong (30 out of 30 VCN or CN samples attached and the strength of adhesion is greater than the strength of the retina, resulting in tearing of the retina upon attempts to detach mechanically), However, in all cases attachment is reversible with dilute plasmin [20]. During our *in vivo* experiments two out of four rabbits showed strong attachment of the VCN samples to the retina. Some possible reasons why the other two showed no attachment are: 1. The vitrectomy was not performed properly so that the integrins were not properly exposed for disintegrin attachment. 2. During implantantion, disintegrins could have been in contact with blood. Blood or other fluid could contaminate the surgical field preventing direct contact of the implant to the retinal surface. Future experiments will be performed to perfect the *in vivo* techniques.

Silicone has excellent mechanical and biocompatibility properties, which makes it an excellent substrate for interfacing with soft tissue like the retina. This study demonstrated a technical approach for functionalizing the surface of silicone to promote adhesion, a capability that could be very advantageous in future medical devices. This process was shown in the retina, but may be applicable to brain cortex, spinal cord and different areas of the body where sutures are not an option. Further, this procedure could be adapted for drug delivery in future modifications.

## Figures and Tables

**Table 1:** An ATL Promaster Excimer Laser was used during all the experiments performed. Shown in this table are settings that produced a consistent debris field area on the silicone surface, thus were used during all the experiments reported here.

ATL ProMaster Excimer Laser Settings	
Pattern	Ring Structure
Electron beam power	10 KeV ≤
Pressure	6500 ≤
Pulse rate	150 Hz
Geometrical Shape	Circle
Beam size (Circle)	250 μm
Space between circles	250 μm
Number of turns	5
Overlap	5
Repeat Rate	50 Hz

**Table 2:** Seven different types of silicones were lased and treated with disintegrins to evaluate the degree of disintegrin binding. Samples were evaluated for growth of OVCAR3 by visual inspection. Increase in fluorescence means VCN-silicone supports cell growth which it is proportional to disintegrin binding. **Note:** −−−− indicates no observable cell growth; + through ++++ indicates increasing levels of OVCAR-3 cells growth (increase in green fluorescence).

Cell Growth/Disintegrin Binding Testing		
	CN	VCN
DowCor WL5150	−−−−	−−−−
Nusil MED 4286	−−−−	−−−−
Nusil MED 4810	−−−−	−−−−
Nusil MED 4830	+	+
Nusil MED 4840	++	++
Nusil MED 4850	+++	+++
Nusil MED 4860	++++	++++

**Table 3:** *In vitro* experiments in cadaveric pig eyes were performed to study disintegrin-retina attachment. Four different types of silicones and two different types of disintegrins were used during these experiments. Attachment of silicone samples were assessed with two different disintegrins. −−−− indicates no observable binding; + through ++++ indicate increasing levels of binding.

Disintegrin-Retina Attachment *In vitro*		
	CN	VCN
Nusil MED 4830	−−−−	−−−−
Nusil MED 4840	+	+
Nusil MED 4850	++	++
Nusil MED 4860	++++	++++

**Table 4:** Different sterilization protocols were tested to ensure viability of the disintegrin after sterilization. Group 1 went through the lasing process without any sterilization method. Group 2 was sterilized before the lasing process. Group 3 was sterilized after the lasing process and Group 4 is a control group, silicones were not lased and placed in PBS. −−−− indicates no observable binding; + through ++++ indicate increasing levels of binding.

Sterilization Protocols / Disintegrin-Retina Attachment							
	NonSterile (Group 1)		Control (Group 4)	Sterilization before lasing (Group 2)		Sterilization after lasing (Group 3)	
	CN	VCN	PBS	CN	VCN	CN	VCN
MED 4830	−−−−	−−−−	−−−−	−−−−	−−−−	−−−−	−−−−
MED 4840	+	+	−−−−	+	+	−−−−	−−−−
MED 4850	++	++	−−−−	++	++	−−−−	−−−−
MED 4860	++++	++++	−−−−	++++	++++	−−−−	−−−−

**Table 5:** *In vivo* experiment; Attachment of sterile silicone samples with disintegrins. − indicates no observable attachment or infection; + indicates attachment or infection; × indicates data point was not acquired. At, attachment, In, infection. 1 week (1 w), 2 weeks (2 w), 5 weeks (5 w), 2 months (2 m), 3 months (3 m) indicates the data points where rabbits were evaluated. Rabbit 1(R1) and Rabbit 2(R2) showed attachment during the period they were observed and no signs of infection during the same period. Rabbit 3 (R3) and Rabbit (R4) showed neither attachment nor sign of infection during the period they were maintained.

*In vivo* Experiments / Disintegrins-Retina Attachment										
	1 w		2 w		5 w		2 m		3 m	
	At	In	At	In	At	In	At	In	At	In
R1	+	−	+	−	+	−	+	−	×	×
R2	+	−	+	−	+	−	+	−	+	−
R3	−	−	−	−	−	−	−	−	×	×
R4	−	−	−	−	−	−	−	−	×	×

## Abbreviations:

AMD: Aged Related Macular Degeneration
RP: Retinitis Pigmentosa
IOP: Intraocular Pressure
RGD: Arg-Gly-Asp
CN: Contortrostatin
VCN: Vicrostatin
PBS: Phosphate Buffered Saline
GFP: Green Fluorescent Protein
EtO: Ethylene Oxide
PVD: Posterior Vitreous Detachment
OCT: Optical Coherence Tomography
SEM: Scanning Electron Microscope; FA: Fluorescence Angiography
